# Upadacitinib for the treatment of refractory bullous pemphigoid: a case report and literature review

**DOI:** 10.3389/fimmu.2026.1866166

**Published:** 2026-09-22

**Authors:** Fangying Su, Guanjing Wei, Qunshi Qin, Tai Wang, Minmin Jiang, Zhi Xie

**Affiliations:** Department of Dermatology and Venereology, The People’s Hospital of Guangxi Zhuang Autonomous Region, Guangxi Academy of Medical Sciences, Nanning, China

**Keywords:** autoimmune bullous diseases, bullous pemphigoid, dupilumab, janus kinase(JAK) inhibitors, upadacitinib

## Abstract

Bullous pemphigoid (BP) is a common autoimmune subepidermal blistering disorder that mainly affects elderly individuals aged ≥70 years. Systemic glucocorticoids serve as first-line treatment, and immunosuppressants or biological agents such as dupilumab are added for patients with suboptimal responses. However, a proportion of patients exhibit suboptimal responses or cannot sustain therapeutic benefits under these standard therapeutic regimens, representing an important unmet clinical need. We describe a 74-year-old patient with refractory BP who did not achieve adequate disease control despite sequential treatment with methylprednisolone, mycophenolate mofetil, cyclosporine, and dupilumab. The addition of upadacitinib resulted in substantial clinical improvement and durable disease stability. We also examine existing literature concerning salvage therapeutic options for refractory BP and the real-world application of upadacitinib in this disease. As this is a single-case observation, our work intends to offer clinical insights for the management of difficult-to-treat BP.

## 1 Introduction

Bullous pemphigoid (BP) is the most common autoimmune subepidermal blistering disease, caused by the presence of autoantibodies targeting components of the skin basement membrane region, mainly BP180 and BP230 (1). The resulting immune response initiates an inflammatory cascade, leading to the development of tense blisters and severe pruritus that severely impair the patients’ quality of life. Current treatment strategies are centered on systemic glucocorticoids as the cornerstone therapy. Nevertheless, long-term administration is frequently associated with severe adverse effects, particularly in the elderly, who are more susceptible to complications such as osteoporosis, hyperglycemia, and infections (2).

In recent years, the emergence of biologics and small-molecule targeted therapies has revolutionized the treatment landscape of BP. Dupilumab, a monoclonal antibody targeting interleukin-4 receptor α (IL-4Rα), has demonstrated efficacy in a large proportion of patients with BP by suppressing type 2 T-helper (Th2) immune responses (3, 4). However, we observed that a subset of patients still exhibits an inadequate response to dupilumab therapy, and experiences disease recurrence during follow-up despite receiving standardized therapy (5, 6).

Janus kinase (JAK) inhibitors, which modulate the Janus kinase/signal transducer and activator of transcription (JAK/STAT) signaling pathway, have garnered increasing attention in the management of diverse autoimmune diseases. Upadacitinib, a selective JAK1 inhibitor, has shown promising efficacy in conditions including rheumatoid arthritis, atopic dermatitis, and psoriasis by interfering with proinflammatory cytokine signaling (7, 8). However, its role in the treatment of BP remains underexplored. Although a small number of case reports have documented successful use of upadacitinib in BP (9–11), clinical data are still limited. This case report describes a patient with recurrent BP who was refractory to treatment with methylprednisolone, mycophenolate mofetil, dupilumab, and cyclosporine, but achieved long-term disease stability with significant efficacy after Upadacitinib therapy. We also performed a comprehensive literature review to clarify its potential mechanism of action and clinical significance.

## 2 Case presentation

### 2.1 Chief complaint and history of present illness

A 74-year-old female patient presented with a six-month history of generalized erythema and intractable pruritus, accompanied by new-onset generalized tense blisters for more than one month. The patient initially developed spontaneous widespread erythema and severe pruritus half a year prior, with scratching-induced skin abrasion, erosion, and exudation. Empirical treatments at external hospitals only provided temporary relief, followed by repeated disease flares. One month before admission, tense vesicles and clear-fluid bullae arose on intact lower extremity skin and rapidly generalized, without oral mucosal involvement or systemic symptoms. The patient’s general condition was stable, and she denied a history of chronic diseases.

### 2.2 Physical examination

Vital signs within normal limits; Weight: 50.5 kg. Generalized erythema was observed across the body, with scattered small vesicles and large bullae on the erythematous base. The blister walls were tense and contained clear fluid. Brown crusts were noted in the center of the lesions, surrounded by annular and chrysanthemum-like blisters. The Nikolsky sign was negative. (**Figure 1**).

**Figure 1:**
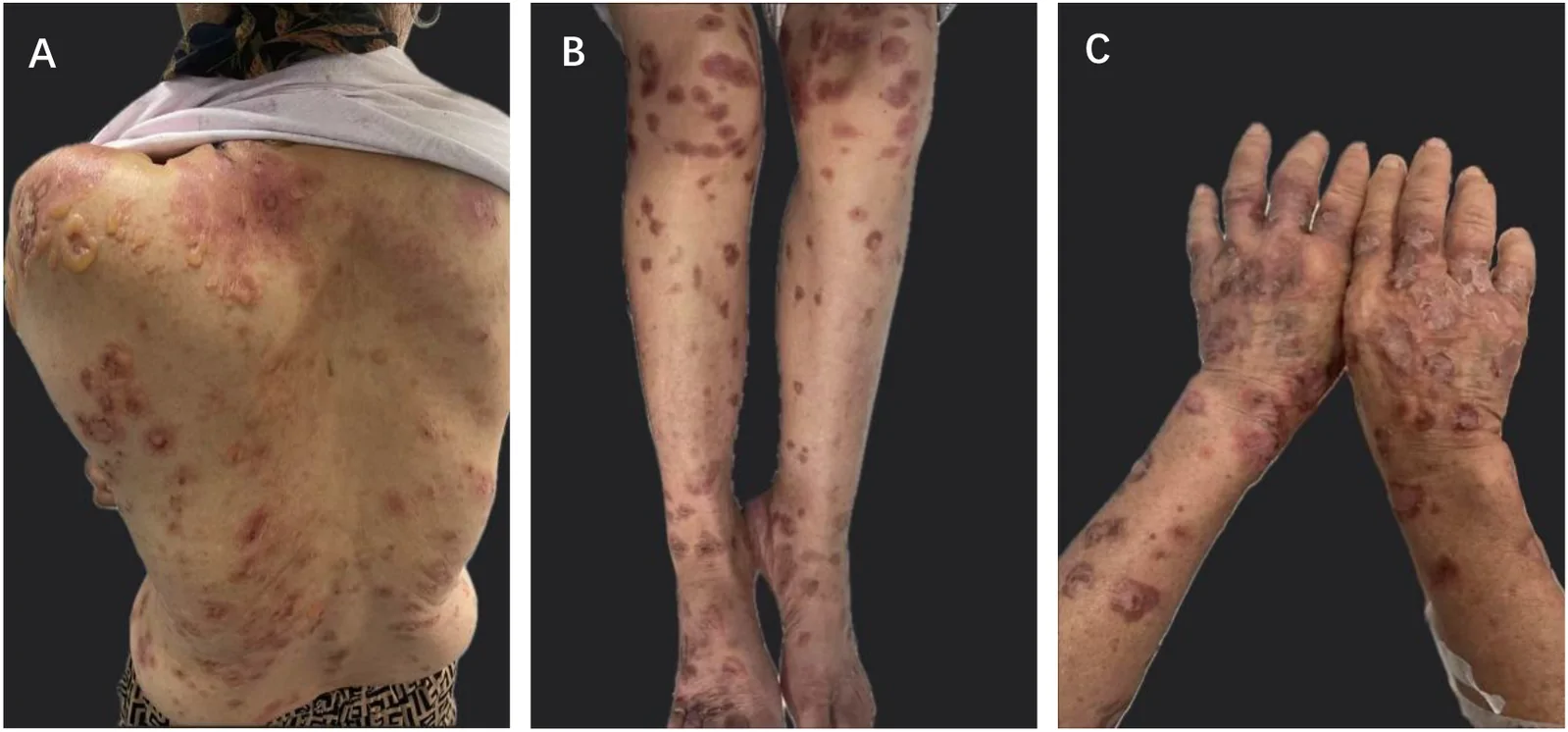
**(A-C)** Clinical features of blistering lesions at initial presentation: Variable-sized vesicles and tense bullae arising from erythematous bases were distributed on the back, lower extremities and forearms. Blisters had tense walls with clear serous fluid, most lesions showed central brown crusting, surrounded by annular, chrysanthemum-like small blister clusters. Nikolsky sign was negative.

### 2.3 Auxiliary examinations

Skin biopsy (right forearm): The specimen showed epidermal atrophy with basket-weave hyperkeratosis. No significant edema was observed in the spinous layer. Subepidermal blisters formed with a small amount of inflammatory exudate in the blister cavity, accompanied by interstitial myxoid degeneration. A moderate number of lymphocytes, plasma cells, and a small number of neutrophils and eosinophils infiltrated around the blood vessels and skin appendages in the superficial dermis. (**Figures 2A, B**).

**Figure 2:**
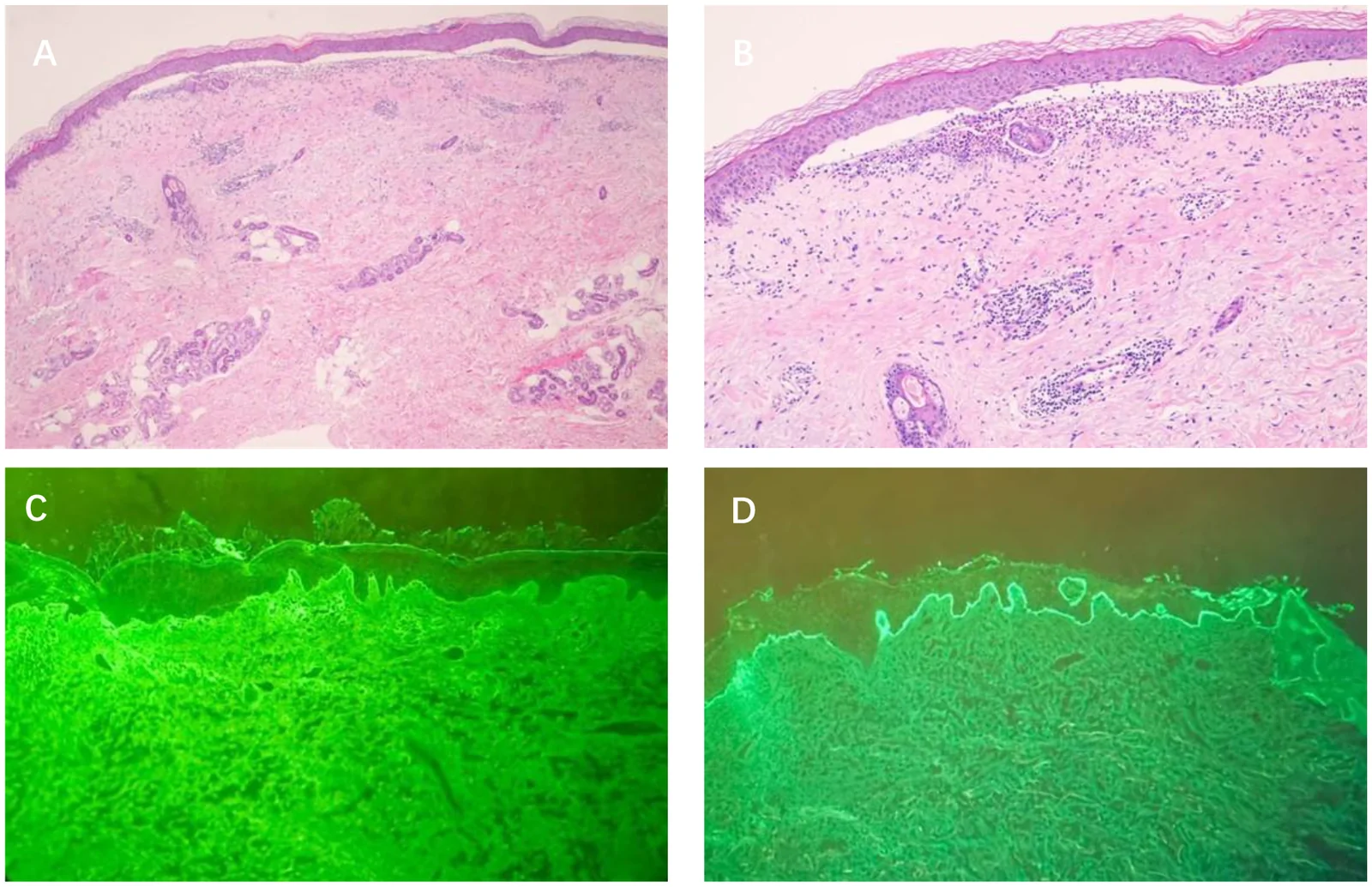
**(A, B)** Light microscopy of a fresh bulla from a patient with BP(hematoxylin-eosin stain): subepidermal blister formation with discrete inflammatory infiltrate (eosinophils and neutrophils) in the blister cavity and in the dermis by light microscopy. **(C, D)** DIF: Linear IgG **(C)** and C3 **(D)** at the dermoepidermal junction.

Direct immunofluorescence: IgG (band-like fluorescence at the epidermal basement membrane), C3 (linear fluorescence at the epidermal basement membrane) (**Figures 2C, D**).

All other examinations were unremarkable. Notably, chest imaging revealed right lung bronchiectasis with latent infection, which posed a potential infectious risk for subsequent immunosuppressive therapy.

### 2.4 Treatment course

The patient's treatment course and response are summarized in **Figure 3**. The comprehensive clinical and pathological findings confirmed the diagnosis of BP (12–14). At admission, the baseline Bullous Pemphigoid Disease Area Index (BPDAI) score was 103, indicating severe disease activity. Initial treatment comprised intravenous methylprednisolone 40 mg once daily (0.8 mg/kg) plus oral nicotinamide, yet new blister formation persisted and symptoms remained uncontrolled. Escalation to intravenous methylprednisolone 60 mg daily (1.2 mg/kg) failed to resolve recurrent new blisters, prompting a regimen shift to intravenous dexamethasone 10 mg daily combined with oral mycophenolate mofetil 1 g twice daily. Thoracic CT revealed bronchiectasis complicated with latent infection. Prophylactic piperacillin-tazobactam was administered to mitigate immunosuppression-related infectious risks.

**Figure 3:**
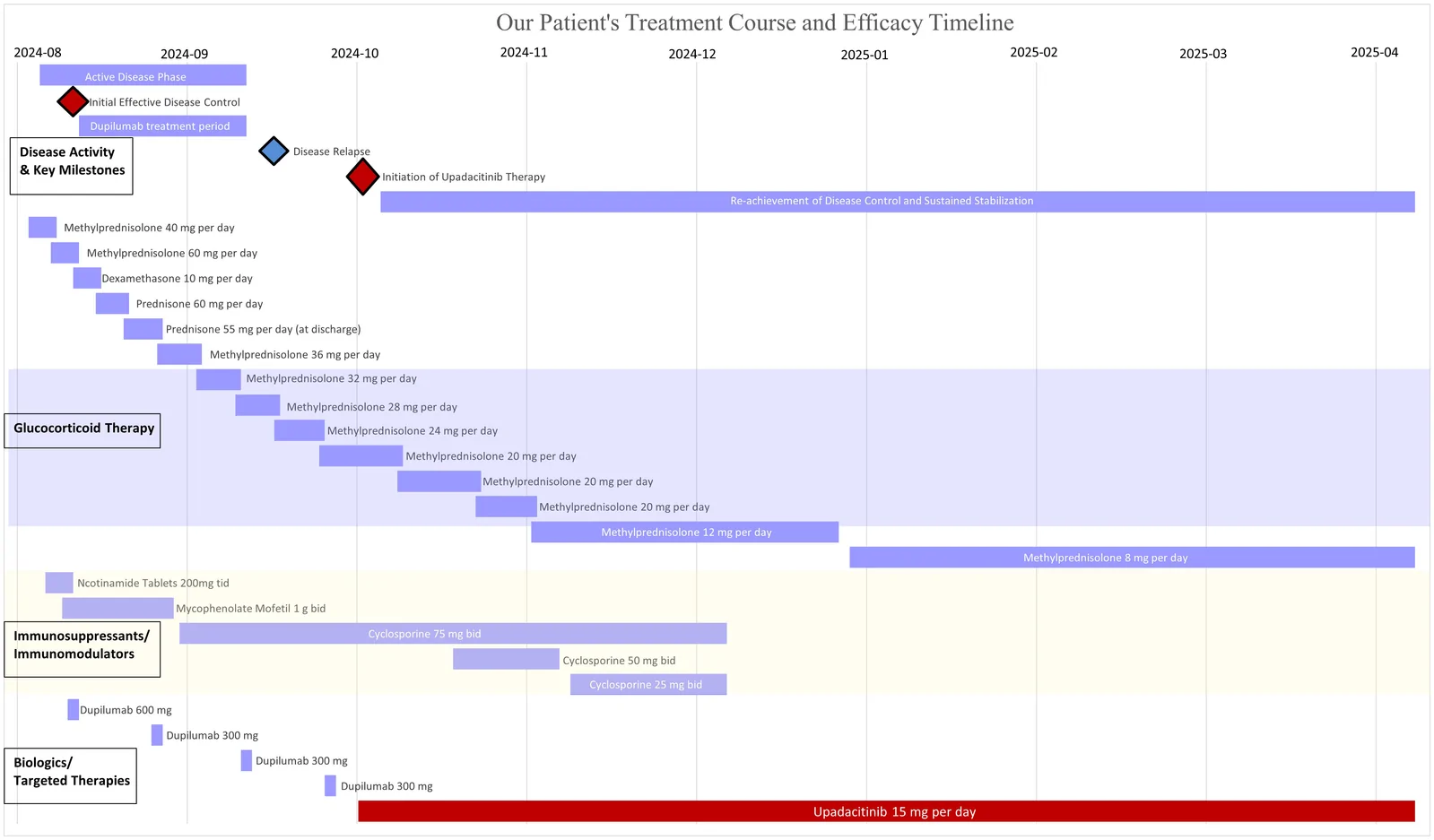
Timeline of clinical management and disease progression for the patient. The horizontal axis represents the time course (from August 2024 to April 2025, in months). Key events include shifts in disease status (active disease, initial control, relapse, sustained stabilization; denoted by diamonds), administration and dose adjustments of glucocorticoids, sequential use of immunosuppressants, and the transition from initial biologic therapy (dupilumab) to targeted therapy (upadacitinib) post-relapse, with bars indicating the duration of each treatment regimen.

Despite this intervention, intractable pruritus and new lower-extremity blisters persisted. Subsequent therapeutic modification retained mycophenolate mofetil, along with adjunctive dupilumab and oral prednisone (1.2 mg/kg). Marked clinical improvement was achieved within 5 days, with blister crusting, relieved itching, a BPDAI score of 75, most pre-existing lesions controlled, and no new lesions, allowing hospital discharge. A tapering glucocorticoid regimen was prescribed at discharge (the planned tapering schedule: reduce the total dose of methylprednisolone by 10% every 7 days for the initial 3-4 weeks, followed by dose adjustments every 2-4 weeks based on clinical disease activity).

Stable symptom control was sustained at the one-week follow-up. New blister relapse emerged two weeks later, prompting medication adjustment: methylprednisolone dose was reduced, and cyclosporine was introduced while mycophenolate mofetil was kept. Two weeks later at follow-up, continued resolution of blister and erythema was observed, with partial fading of hyperpigmentation and a BPDAI of 37, indicating subsiding disease activity; mycophenolate mofetil was withdrawn. Methylprednisolone was gradually tapered stepwise during successive follow-ups. No deterioration of pulmonary infection was detected throughout the administration of cyclosporine and mycophenolate mofetil.

Disease relapse occurred 2 weeks later, presenting as new pruritic blisters on both lower legs; the BPDAI score rebounded to 48, suggesting renewed disease activity. At that time, the methylprednisolone had been tapered to 20 mg daily as scheduled (equivalent to prednisolone 0.5mg/kg). We initially evaluated multiple alternative therapeutic options, including oral adjuvant agents (doxycycline, dapsone), rituximab, and omalizumab. However, long-standing disease duration, latent pulmonary infection, and limited financial capacity disqualified these regimens for our patient, prompting us to seek other affordable, effective, and clinically feasible salvage alternatives.

We reviewed the literature and found that the JAK/STAT pathway is involved in the immunopathogenesis of BP (15). Based on documented clinical evidence supporting the efficacy of upadacitinib in BP (9, 10), as well as our team’s previous successful experience in treating patients with BP with upadacitinib (11)(**Table 1**), we added oral upadacitinib 15 mg daily on the basis of the patient’s stable existing doses of methylprednisolone and cyclosporine. Baseline workup before treatment—including complete blood count, inflammatory markers (CRP/ESR), tuberculosis screening, viral serology, and chest CT—showed no significant abnormalities. Concomitant supportive therapies including alfacalcidol for calcium supplementation and rabeprazole sodium for gastrointestinal protection were administered, alongside amphotericin B mouth rinses for candida prophylaxis.

**Table 1:** Summary of clinical studies on selective JAK1 inhibitors (upadacitinib, abrocitinib) for BP.

Cases	JAK1 inhibitor	Sample size	Dosing regimen	Prior failed therapies	Key efficacy outcomes	Safety profile
Nash D et al., 2023 (10)	Upadacitinib	1 (81-year-old female)	15 mg qd; steroid tapered in 20d	Systemic steroid monotherapy (poor response)	Complete resolution of cutaneous lesions and pruritus at 2 months; no new blisters observed at 5-month follow-up	No treatment-related adverse events reported
Gresham LM et al., 2023 (9)	Upadacitinib	1 (74-year-old male with immunotherapy-induced drug-related BP)	15 mg qd	High-dose steroid (6w persistent new blisters)	Marked reduction in blisters and urticarial eruptions within 4 weeks	No serious treatment-related adverse events; patient deceased due to underlying tumor progression
Jiang W et al., 2024 (35)	Abrocitinib	2 (52-year-old female, 83-year-old male)	100 mg qd	Steroid, cyclosporine, omalizumab (recurrence)	Significant reduction in anti-BP180 autoantibodies at 1 month; complete cutaneous clearance, rapid glucocorticoid tapering	No adverse events reported during 2–5 months of follow-up
Su F et al. , 2024 (11)	Upadacitinib	1 (66-year-old male)	15 mg qd	Steroid + dupilumab (new-onset psoriasis)	Near-complete resolution of both BP and psoriasis lesions at 10 weeks; significant reduction in BPDAI score	No thromboembolic events, infections, or other JAK inhibitor-related adverse events observed
Chen Y et al , 2025 (36)	Abrocitinib	43 (21 in abrocitinib group, 22 in azathioprine control group)	Methylprednisolone + 100 mg qd	Steroid, conventional immunosuppressants (inadequate control)	New lesion control achieved within 7 days; shorter time to minimum maintenance therapy, significantly lower total glucocorticoid exposure; 36-week complete remission rate 52.4% vs 9.1% in control group	Mild transient hyperglycemia and hyperlipidemia only; no grade 3–4 serious adverse events, no thromboembolic events reported
Hu Y et al. , 2026 (37)	Abrocitinib	1 (58-year-old male with alcoholic liver cirrhosis)	100 mg qod + topical steroid	Systemic steroid (limited effect)	Marked pruritus improvement at 2 weeks; complete resolution of both BP and psoriasis lesions at 24 weeks; no recurrence during 8-month follow-up	No gastrointestinal adverse events, infections, or liver function abnormalities reported

Overview of clinical studies on the efficacy and safety of two currently available selective JAK1 inhibitors (upadacitinib and abrocitinib) for BP. The table summarizes the study design, therapeutic regimens prior to JAK1 inhibitor initiation, core efficacy endpoints, and safety profiles. Available case-based data suggest that these selective JAK1 inhibitors may rapidly relieve pruritus and improve blistering lesions, and may facilitate glucocorticoid tapering with acceptable short-term tolerability among elderly patients with comorbidities. However, evidence remains limited, and further larger-scale studies are warranted.

Obvious regression of lower limb lesions and complete cessation of new blisters were observed two weeks after upadacitinib administration (**Figures 4A–C**). The BPDAI score decreased to 36. At the four-week follow-up, further lesion resolution was observed, with near-complete crusting of pre-existing blisters, absence of new blisters, and a BPDAI score of 29, indicating a stable disease state (**Figures 4D–F**). During subsequent follow-ups, upadacitinib was maintained at 15 mg once daily, cyclosporine was gradually tapered and discontinued, and methylprednisolone was slowly reduced to 8 mg once daily. The patient remained stable without recurrence during the 4-month follow-up period. Repeat assessments at follow-up, including complete blood count, inflammatory markers (CRP/ESR), viral serology and chest CT, revealed no significant abnormalities.

**Figure 4:**
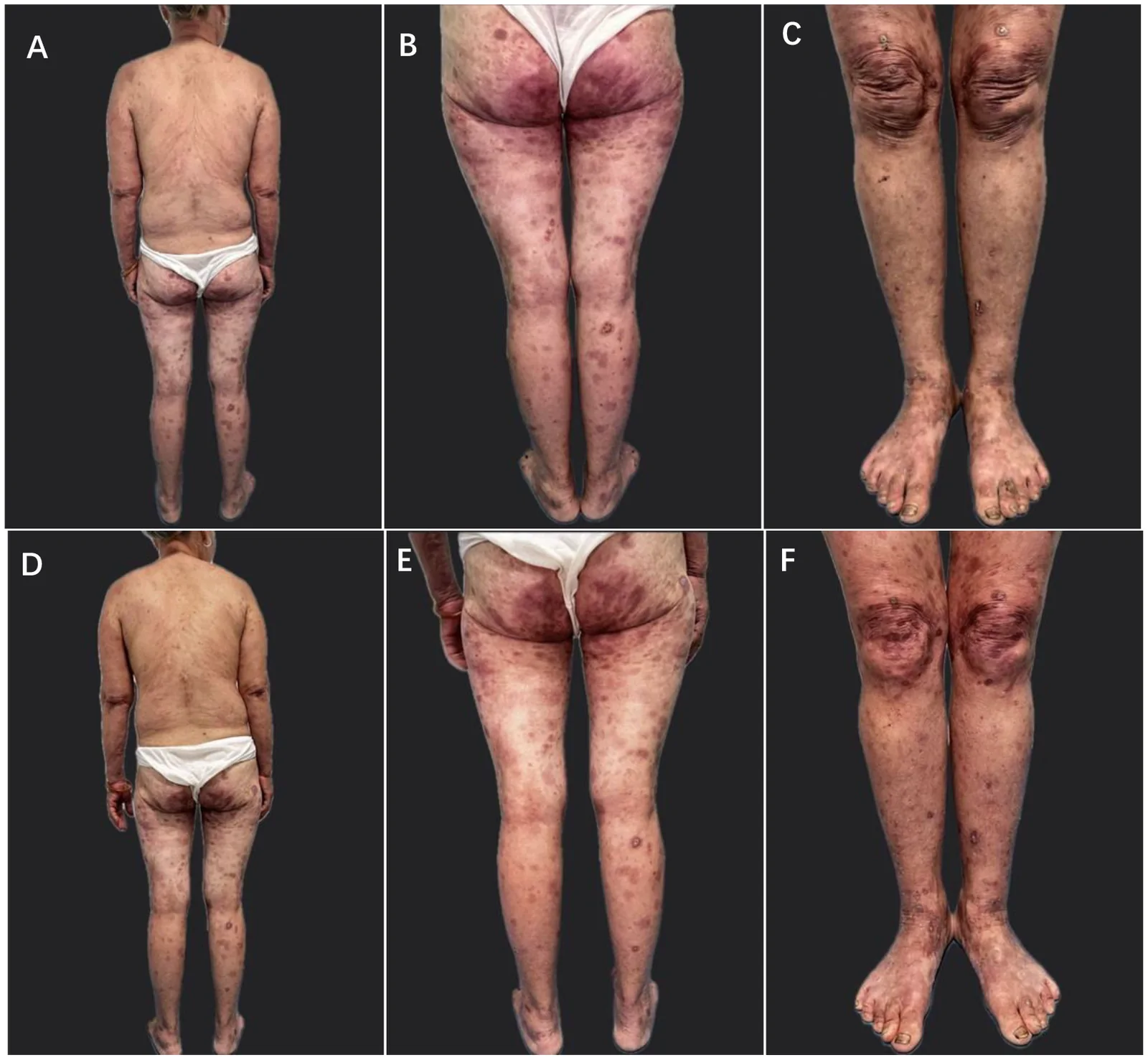
**(A-C)** Clinical images of the trunk and lower limbs after 2 weeks of upadacitinib treatment: lower extremity blisters began to dry and crust, with no new blister formation, and focal pigmentation was observed. **(D-F)** Clinical images of the trunk and lower limbs after 4 weeks of upadacitinib treatment: Lower extremity blisters resolved completely with no new lesions, and only residual postinflammatory hyperpigmentation was present.

Notably, serum BP180/230 autoantibody titres and formal pruritus numerical rating scale scores were not prospectively collected for this retrospective case, which represents an important limitation of the present report. Serial BPDAI scores were therefore used as the primary objective metric to evaluate disease activity and treatment response throughout the clinical course.

## 3 Discussion

### 3.1 The role of dupilumab in the treatment of BP

Dupilumab is a fully human monoclonal antibody that selectively binds to the IL-4 receptor α subunit, simultaneously inhibiting the signaling of IL-4 and IL-13—key cytokines driving the Th2 immune response (6). As a representative biologic agent for type-2 inflammation-mediated diseases, dupilumab has been widely studied and applied in BP (16). Multiple clinical studies and case series have confirmed that dupilumab can effectively control skin inflammation, reduce blister formation, relieve pruritus, and lower systemic glucocorticoid dependence in patients with BP, demonstrating a favorable safety profile. It has become an important second-line treatment option for those who are intolerant to or have a poor response to glucocorticoids and traditional immunosuppressants (17). Therefore, we implemented combination therapy with dupilumab due to the inadequate response to initial immunosuppressive treatment. In our case, the patient still developed recurrent disease activity during glucocorticoid tapering despite this therapeutic intervention. Consistently, clinical evidence demonstrates that approximately 10%-20% of patients with BP are primary- or secondary-refractory to dupilumab, which may be related to the heterogeneity of disease pathogenesis, the involvement of non-Th2 inflammatory pathways, and individual differences in cytokine profiles (5). These findings collectively highlight the need to explore alternative therapeutic strategies.

### 3.2 Alternative treatment strategies for BP and case-specific assessment

We reviewed relevant literature to identify alternative therapeutic regimens for refractory bp.

Rituximab, a chimeric anti-CD20 IgG1 monoclonal antibody, induces sustained B-cell depletion lasting 6-12 months to suppress humoral immunity (18, 19). Multiple clinical studies have confirmed its efficacy for refractory BP, and current guidelines recommend it as rescue therapy for patients unresponsive to dupilumab (20, 21). Nevertheless, this patient’s advanced age, chronic immune dysfunction, and latent bronchiectasis with uncertain infectious activity markedly elevated her risk of severe infection.

Omalizumab is an off-label alternative reported for BP, with optimal efficacy in patients with elevated serum anti-BP180-NC16A IgE levels (22). However, our patient had normal total serum IgE, and testing for anti-BP180-NC16A IgE was unavailable at our center. In addition, our clinical team lacks experience administering omalizumab for BP treatment.

Immunoadsorption (IA) is a guideline-endorsed extracorporeal rescue therapy for severe refractory BP (17). A prospective single-center study demonstrated that three consecutive days of IA reduced circulating pathogenic anti-BP180 IgG autoantibodies by an average of 89%. This intervention yielded complete remission rates of 50% at 2 months and 90% at 6 months, while mitigating pruritus and accelerating glucocorticoid tapering (23). IA selectively eliminates autoantibodies from plasma; however, its clinical application is limited by equipment requirements, the need for temporary central venous catheterization, and repeated hospital admissions, which are not feasible in general hospitals. Aside from mild procedural adverse events including hypotension and citrate-induced hypocalcemia, indwelling venous catheters carry life-threatening risks of bloodstream infection, which requires long-term surveillance for infectious signs throughout treatment.

Doxycycline and dapsone are oral steroid-sparing adjuvants. Doxycycline alleviates inflammation by inhibiting lesional matrix metalloproteinases and neutrophilic infiltration, whereas dapsone exerts potent antipruritic and anti-inflammatory effects on neutrophil-rich lesions covered with clustered tiny blisters. The two agents have slow onsets of action, at 8-10 weeks and 4-8 weeks, respectively (12).

Collectively, rituximab, omalizumab and IA are all recognized rescue options for refractory BP, yet they are unsuitable for this patient due to the aforementioned safety hazards and unaffordable treatment costs. By contrast, the oral adjuvants were ruled out for a distinct reason: the patient had six-month disease history and experienced recurrent blister flares during tapering of glucocorticoids, conventional immunosuppressants and dupilumab. Rapid control of cutaneous lesions was urgently needed to prevent extensive generalized relapse, a clinical demand that these slow-acting oral agents could not meet. We therefore turned to search for faster, cost-effective salvage therapy. Notably, IA remains a valuable short-term intervention for patients with fulminant widespread lesions and extremely high autoantibody titers, provided that adequate hospital facilities and financial support are accessible.

### 3.3 Rationale for upadacitinib selection and relevant literature review

The pathogenesis of BP is complex and involves intricate interactions between the immune system and the basement membrane regions of the skin. Autoantibodies, mainly IgG, target BP180 and BP230, leading to complement activation, inflammatory cell recruitment, and subsequent basement membrane damage, ultimately resulting in blister formation (1). Th2 cells play a pivotal role in this process by secreting cytokines such as IL-4, IL-5 and IL-13, which promote eosinophil infiltration and autoantibody production (24, 25).

Studies have demonstrated that the expression of JAK/STAT proteins is upregulated in the skin lesions of patients with BP, indicating that the JAK/STAT signaling pathway may be implicated in the pathogenesis of BP-associated cutaneous lesions (26). JAK inhibitors, as a novel class of small-molecule targeted agents, have attracted extensive attention in recent clinical research and are increasingly recognized as a valuable therapeutic alternative for autoimmune bullous diseases—particularly BP refractory to conventional therapies (27). In recent years, a variety of JAK inhibitors have been applied clinically, and selective JAK inhibitors are recommended for the treatment and efficacy evaluation in pemphigoid diseases (28–30). JAK1 occupies a central position in transducing multiple cytokines critical for BP immunopathogenesis, including IL-4, IL-5, IL-13, and interferon-γ (IFN-γ) (31). These cytokines drive eosinophil recruitment, Th1/Th2 immune skewing, and local tissue injury in pemphigoid skin. Therefore, selective inhibition of JAK1 can simultaneously block both Th2- and IFN-γ-mediated inflammatory cascades that contribute to blister formation.

Tofacitinib, a first-generation non-selective JAK1/JAK3 inhibitor, has shown therapeutic benefits across several published refractory BP case series. Nevertheless, one notable case report documented acute bilateral hemiretinal vein occlusion shortly after tofacitinib initiation, with alternative etiologies largely excluded, raising a potential drug-related safety concern. Though most reported cases tolerated tofacitinib well, this adverse-event observation underscores the necessity for individualized risk stratification and close clinical surveillance when deploying this agent, especially among elderly patients with BP burdened with multiple comorbidities (32).

As another member of the JAK family, tyrosine kinase 2 (TYK2) participates in the signaling pathways downstream of type-1 interferons, IL-12, IL-23 and IL-10, triggering immune responses distinct from those mediated by JAK1, JAK2 and JAK3 (33). The currently approved TYK2 selective inhibitor deucravacitinib is indicated for moderate-to-severe plaque psoriasis and psoriatic arthritis, and its therapeutic potential in systemic lupus erythematosus and dermatomyositis has attracted considerable attention. Nevertheless, there is no clinical evidence supporting the use of TYK2 inhibitors for BP treatment. Of note, one published case of deucravacitinib-induced BP has been reported, presumably resulting from disrupted Th1-Th2 immune balance following TYK2 inhibition (34). This finding makes us exercise greater caution when considering TYK2 inhibitors as a therapeutic option for refractory BP.

As a highly selective second-generation JAK1 inhibitor, upadacitinib preferentially interferes with JAK1-dependent cytokine signaling while minimizing perturbation of JAK2- and JAK3-mediated pathways. This pharmacological profile theoretically reduces off-target effects and may confer particular suitability for older patients with BP with underlying comorbidities, representing a mechanistic rationale for its off-label use in this setting.

A systematic literature search identified favorable therapeutic responses to the selective JAK1 inhibitors abrocitinib and upadacitinib in the management of BP (**Table 1**). In addition, our institutional experience with upadacitinib in patients with BP further suggests that this drug class may represent a promising therapeutic alternative for those refractory to conventional treatment regimens or ineligible for first-line biologic agents. As noted above, the JAK/STAT pathway is involved in BP immunopathogenesis, and JAK1 inhibition may suppress the autoimmune inflammation driving BP cutaneous lesions.

After excluding active tuberculosis and progressive pulmonary infection, we added oral upadacitinib 15 mg daily as salvage therapy on the basis of stable doses of methylprednisolone and cyclosporine. Consistent with previous reports, JAK inhibition rapidly controlled symptoms within two weeks, resolving intractable pruritus and recurrent lower-extremity blisters. This strategy allowed gradual tapering and discontinuation of cyclosporine, with methylprednisolone successfully reduced to a low maintenance dose of 8 mg daily. During the subsequent four-month follow-up maintenance phase, the patient received oral upadacitinib 15 mg once daily combined with methylprednisolone 8 mg once daily. Supportive therapies including calcium supplementation, gastrointestinal protection and candida prophylaxis were administered concomitantly. The patient maintained sustained remission throughout this period, without disease relapse, secondary infection, or any adverse events such as thromboembolism.

### 3.4 Limitations and publication-bias considerations

Almost all current clinical evidence for JAK-inhibitor use in pemphigoid diseases originates from case reports and small retrospective case series, rather than randomized controlled trials. An important concern in this body of evidence is publication bias: cases demonstrating favourable therapeutic responses are far more likely to get published, whereas treatment failures, partial responses, and drug-related adverse events are frequently left unreported. As a result, the existing literature may overestimate real- world efficacy and underestimate potential safety risks of JAK inhibitors for BP.

The present report has several inherent limitations. First, this is a single retrospective case observation with only 4-month of follow-up; long-term efficacy and safety cannot be inferred from this individual patient. Second, we lacked prospective serial measurements of anti-BP180/BP230 autoantibody titres and formal pruritus numerical rating scale scores, relying exclusively on BPDAI as the outcome measure. Third, upadacitinib was used off-label for BP; no phase II/III prospective trial data are available to define optimal dosing, timing of combination glucocorticoid tapering, or long-term risk-benefit profiles in BP populations. Accordingly, larger-scale, preferably prospective clinical studies are required to further validate the efficacy and safety of JAK1-selective inhibitors for refractory BP. Clinicians should perform individualized risk-benefit assessments before initiating upadacitinib and maintain rigorous long-term laboratory and clinical surveillance throughout treatment.

## 4 Conclusion

In conclusion, this individual patient with refractory BP failing conventional glucocorticoids, classic immunosuppressants and dupilumab, the addition of upadacitinib was associated with rapid clinical improvement, enabling further tapering of concomitant immunosuppressive agents and sustaining disease stability during short-term follow-up. Accumulating isolated case reports, together with our present observation, point to the potential value of JAK1-selective inhibitors for refractory BP. Nevertheless, multiple guideline-recommended salvage interventions are subject to practical constraints including safety concerns and limited accessibility for routine-use. Upadacitinib may serve as a feasible salvage therapeutic option for carefully selected patients with treatment-resistant BP, yet generalized conclusions cannot be derived from case-based evidence alone. Therefore, the efficacy of upadacitinib for BP still requires further verification in larger-scale clinical trials, and its underlying immunological mechanisms warrant further mechanistic study.

## Data Availability

The original contributions presented in the study are included in the article/supplementary material, further inquiries can be directed to the corresponding author/s.
